# The Role of Cyclin-Dependent Kinase 4/6 Inhibitors Treatment in Oligometastatic Breast Cancer: A Case Report on a Possible Curative Intent Strategy

**DOI:** 10.7759/cureus.34893

**Published:** 2023-02-12

**Authors:** Ana Valente, Nuno Teixeira Tavares, Cláudia Caeiro, Miguel Barbosa, Isabel Augusto

**Affiliations:** 1 Medical Oncology, Centro Hospitalar e Universitário de São João, Porto, PRT

**Keywords:** palbociclib, cdk 4/6 inhibitors, metastasectomy, locoregional treatment, metastatic breast cancer, oligometastatic breast cancer

## Abstract

A small but important subset of patients with metastatic breast cancer has an oligometastatic disease. Some of these patients are highly responsive to systemic therapy and have the potential to achieve complete remission with treatment. However, it remains to be clarified the best locoregional and systemic treatment strategy for such patients and what features can determine whose patients are the best candidates. We also don’t know what will be the role of cyclin-dependent kinase 4/6 inhibitors in those cases. We report the case of a 41-year-old woman with HR-positive/HER2-negative oligometastatic breast cancer who, after an excellent response to systemic treatment with palbociclib, anastrozole, and goserelin, underwent breast surgery and liver metastasectomy. After completing three years of systemic treatment, the CDK inhibitor was discontinued, maintaining the hormone therapy. The patient remained under regular follow-up with no evidence of disease after eight months.

## Introduction

Metastatic breast cancer (MBC) has long been considered an incurable condition. Usually, locoregional (LR) or systemic treatments have a palliative intent. However, an emerging subset of these patients has low-burden metastatic disease, in some cases defined as oligometastatic (OM) disease. OM breast cancer is considered an intermediate state between localized disease and widespread metastatic disease, as first described by Hellman and Weichselbaum [1]. It is also defined by some authors as the presence of up to five metastatic lesions, with a potential for cure with local treatment [2-4].

Nonetheless, there is no standard definition of oligometastatic disease. Around 20% of the patients with MBC have OM disease and this number is expected to increase in the upcoming years due to significant improvements in diagnostic techniques and the increasingly effective locoregional and systemic treatments [2]. Evident interest in this entity is emerging, but many questions remain unanswered.

The data about LR treatments (for primary breast tumors and metastatic lesions) and systemic treatments is not consensual, and it mainly results from retrospective studies. Some have also reported prognostic factors for patients with OM disease based on retrospective data [5]. However, it needs to be clarified which is the most appropriate therapeutic approach and whose patients are the best candidates. We discuss the management of a patient with de-novo hormone receptor (HR) +/ human epidermal growth factor receptor 2 (HER2) - oligometastatic breast cancer (OMBC), treated with Palbociclib, Hormone therapy (HT), and subsequent combined surgical approach, with excellent outcomes. 

## Case presentation

**Figure 1:**
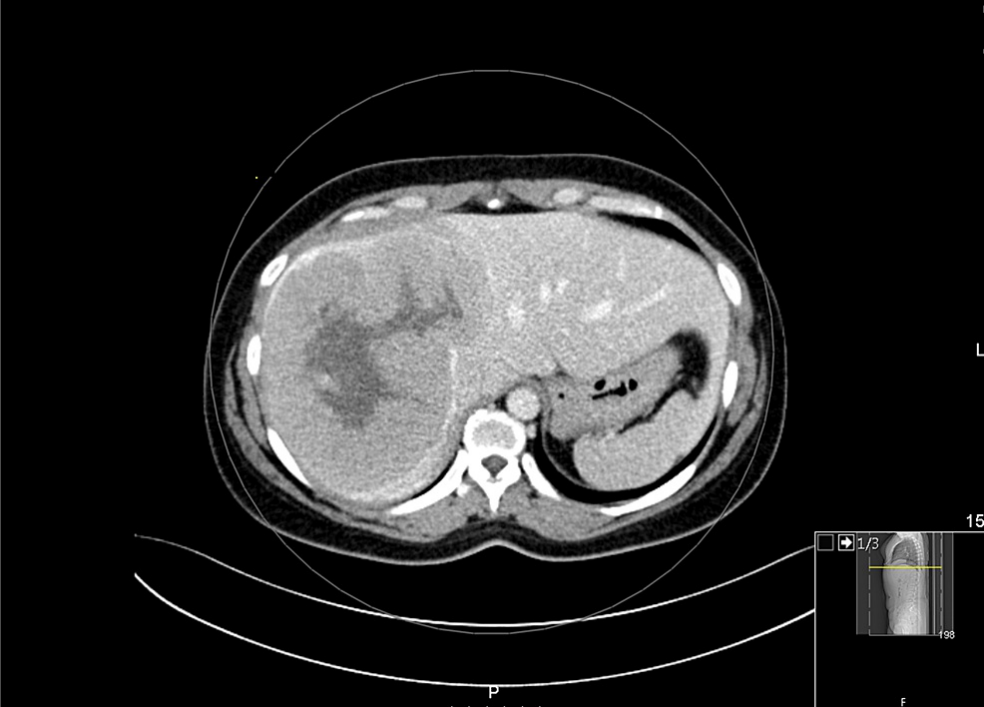
Abdominal CT scan showing a heterogeneous lesion, centered in the right lobe of the liver, measuring 12x16 cm, with hypodense central areas, suggesting some necrotic component.

In January 2018, a 41-year-old woman, with no relevant comorbidities or family history, started with abdominal pain in the right hypochondrium. Physical examination revealed hepatomegaly, and an abdominal ultrasound (US) showed a suspicious hepatic lesion. An abdominal computed tomography (CT) scan was performed and showed a heterogeneous lesion, in the right lobe of the liver, measuring 12x16 cm (Figure 1). 

**Figure 2:**
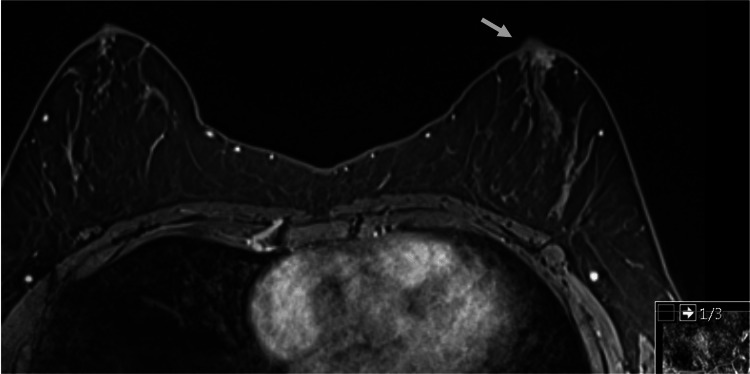
Breast MRI showing a nodular area in the retroareolar region of the left breast, measuring 10 mm.

A core needle biopsy revealed a liver metastasis of primary breast cancer. Immunohistochemistry (IHQ) described hormone receptor (HR) positive (estrogen receptor and progesterone receptor of 100%) and human epidermal growth factor receptor 2 (HER2) negative. The initial assessment with mammogram and breast US showed no lesions (Bi-RADS 0). A breast MRI identified a nodular area, measuring 10 mm, in the retro areolar region of the left breast, without suspicious lymph node disease (Figure 2). 

**Figure 3:**
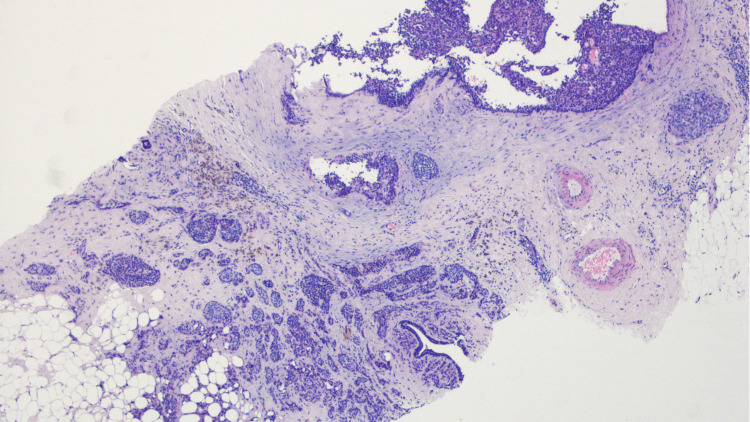
Breast microbiopsy revealed a grade II NST carcinoma, HR positive and HER2 negative, H&E. NST: no special type; HR: hormone receptor; HER2: human epidermal growth factor receptor 2

Subsequently, a second-look breast US was performed and an 8 mm nodular image in the topography of the described nodule was biopsied and marked with a clip. The pathology report demonstrated a grade II no special type (NST) carcinoma, HR-positive and HER2 negative (Figure 3). 

**Figure 4:**
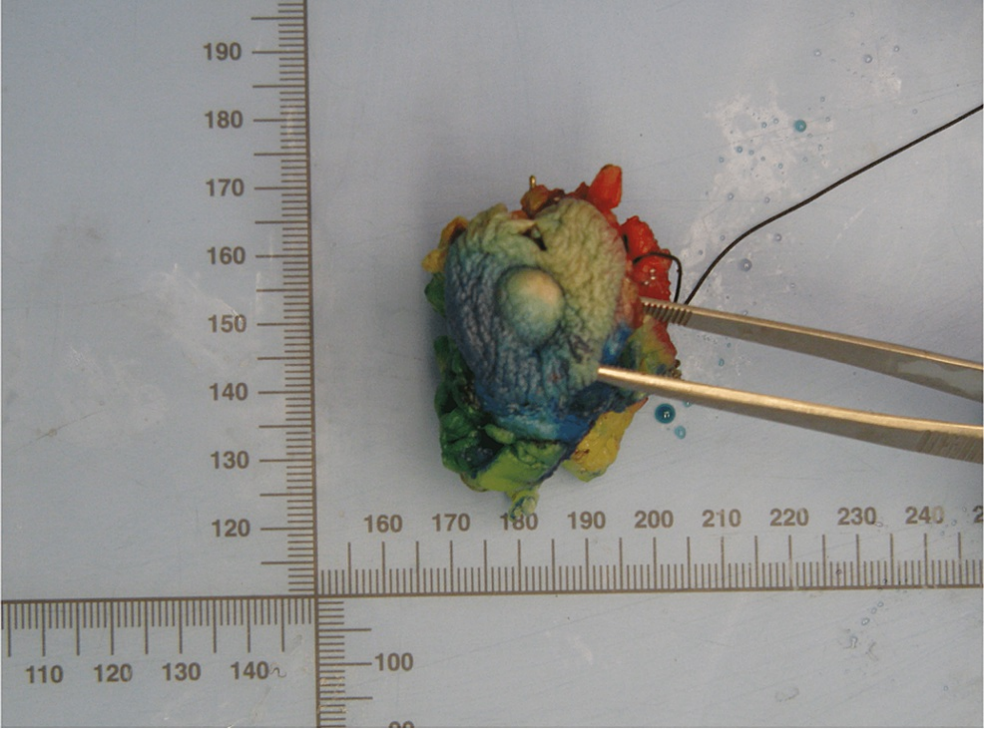
Photo taken after breast surgery: left lumpectomy.

On the Positron Emission Tomography-CT (PET-CT) there was avid F-fluorodeoxyglucose (FDG) uptake in the hepatic metastasis, without other lesions. The clinical case was discussed in a multidisciplinary tumor board, and the patient started systemic treatment with Palbociclib, Anastrozole, and Goserelin. After three cycles of treatment, breast MRI showed a partial response with a significant reduction of the breast lesion, with a residual linear area of 7 mm without a nodular component. Furthermore, an abdominal CT scan presented a partial response, with a hepatic lesion measuring 7x6 cm (reduction > 50%). After a multidisciplinary discussion, it was decided to perform breast surgery and delayed hepatic resection of the metastatic lesion. In November 2018, the patient underwent a left lumpectomy (Figure 4) and sentinel lymph node biopsy (SLNB).

**Figure 5:**
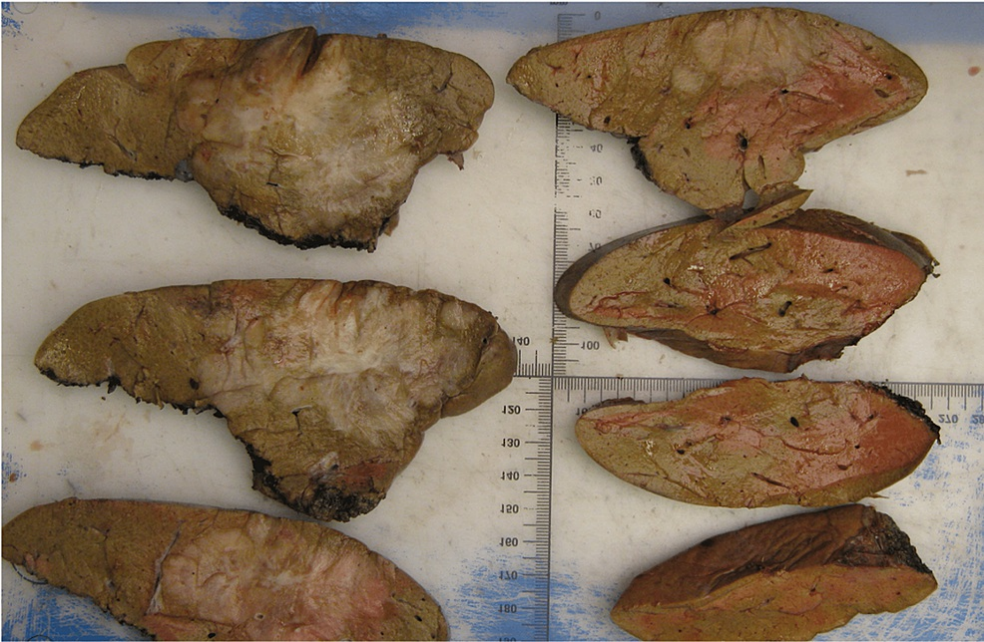
Photo taken after hepatic surgery: right hepatectomy.

After two months, a right hepatectomy was also performed (Figure 5).

**Figure 6:**
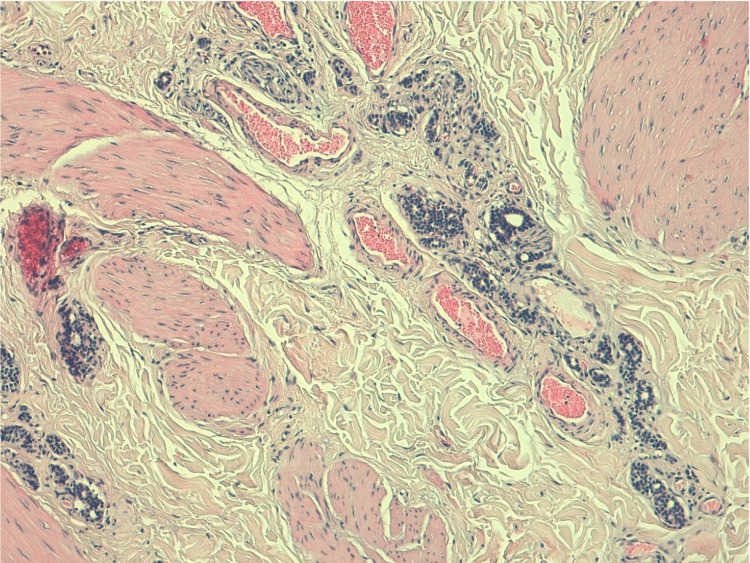
NST breast carcinoma HR-positive/HER2 negative, without lymph node disease (no regression, with more than 50% of residual tumor) - ypT1c N0 M1 (AJCC 8th). NST: no special type; HR: hormone receptor; HER2: human epidermal growth factor receptor 2

**Figure 7:**
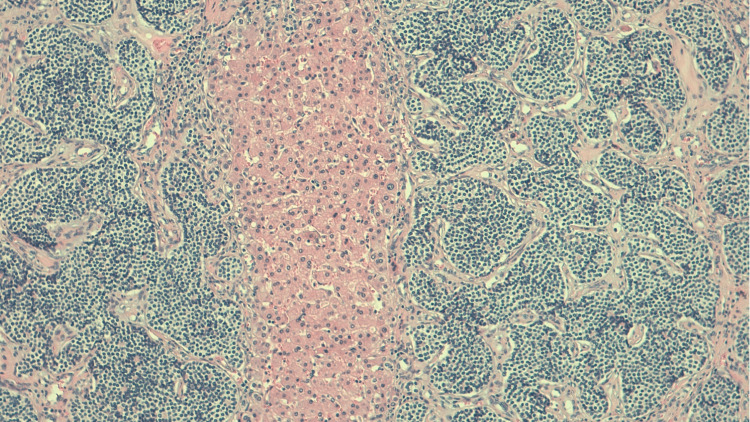
Liver metastasis of primary breast cancer: NST breast carcinoma HR-positive/HER2 negative. NST: no special type; HR: hormone receptor; HER2: human epidermal growth factor receptor 2

Both pathology reports confirmed the presence of NST breast carcinoma HR-positive/HER2 negative (Figure 6, 7), without lymph node disease (no regression, with more than 50% of residual tumor) - ypT1c N0 M1 (AJCC 8^th^).

The case was once again discussed in the multidisciplinary tumor board and it was decided to perform breast “adjuvant” radiotherapy and continue Palbociclib, Anastrozole, and Goserelin. After completing three years of systemic treatment, the CDK inhibitor was discontinued, maintaining the hormone therapy. The patient remains in follow-up without symptoms and is disease-free after eight months of the end of Palbociclib treatment. 

## Discussion

Patients with oligometastatic breast cancer are a subgroup with a better long-term prognosis and may benefit from an aggressive and potentially curative multidisciplinary approach. This approach encompasses LR treatment, directed at the primary tumor and metastatic lesion/lesions, and systemic treatment [2, 5-6]. This type of therapeutic approach must be considered in particular cases. 

Data regarding the tumor biology underlying the process of tumor dissemination and metastasis are not entirely clear due to the impossibility of evaluating all metastatic tissue in patients with advanced breast cancer. However, Miglietta et al., describe two possible scenarios that may underlie tumor progression and metastasis. In the first scenario, the tumor appears as the main source of seeding (called “parental metastasis-to-metastasis dissemination”); in the second, the metastasis itself presents the capacity of a parallel seeding source (“daughter metastasis-to-metastasis dissemination”). This biological behavior is essential to understand the importance of treating the primary tumor and metastatic lesions when the treatment has curative intent. Thus, with a combined approach (LR treatment and systemic therapy) we act by inhibiting the dissemination cascade trigger of tumor cells, as well as reducing the production of tumor clones and the tumor heterogeneity, possibly associated with resistance to future treatments [2].

Some factors provided prognostic information in patients with oligometastatic disease. An international retrospective study with 1200 patients demonstrated that locoregional, systemic therapy and Eastern Cooperative Oncology Group Performance Status (ECOG PS) had 0 prolonged survival outcomes in patients with any breast cancer (BC) subtype. An isolated metastatic site (including liver metastases) was also associated with improved overall survival (OS) in non-triple negative BC [6]. 

One of the most debated areas is the role of primary tumor surgery in de-novo metastatic breast cancer. A recent meta-analysis that included 42 retrospective studies, showed an overall survival (OS) benefit (HR 0,68) for the patients treated with LR therapy (curative resection and radiotherapy of the primary tumor) in metastatic breast cancer [7]. Recently, also four randomized trials evaluated the benefit of primary tumor surgery in patients with MBC. Three of those trials reported no benefit on OS with locoregional treatments (LRT) associated with systemic treatment compared to systemic treatment alone [8, 9, 10]. However, the MF07-01 trial, which included only naïve MBC patients, reported a mortality risk reduction in a five years follow-up, with a median OS of 46 months with LRT versus 37 months in the control group [HR 0,66, 95% confidence interval (CI) 0,49-0,88, p=0,005], particularly in patients with HR+/HER2- BC, age below 55-years-old and solitary bone metastasis [11]. Nonetheless, important questions remain unanswered such as the fact that these trials were not specifically designed for patients with OM breast cancer, some patients presented metachronous metastases (vs. synchronous), locoregional treatment of metastatic lesions was not required and the systemic treatments used are no longer the standard of care. Although these doubts persist, European Society of Medical Oncology guidelines recommends that primary tumor surgery must be considered only in patients with *de novo* stage IV breast cancer with exclusive bone metastases. In other cases, the surgery must be considered for the benefit of the quality of life, after a shared decision with the patient [12].

Patients with OMBC generally have a more favorable prognosis and may also benefit from a more radical approach to metastatic lesions. Therefore, the LR approach to metastatic lesions was similarly assessed in phase II trials. The SABR-COMET trial included 18 patients with OM breast cancer and demonstrated a survival benefit with stereotactic ablative radiotherapy associated with systemic treatment with palliative intent. The five-year OS rate was 42.3% (95% CI, 28% to 56%) for combined treatment vs. 17.7% (95% CI, 6% to 34%) with only standard-of-care systemic treatments [13]. Contrariwise, the phase II/III NRG-BR002 trial evaluated 125 OM breast cancer patients with a controlled locoregional disease and four or fewer metastases. These patients were randomized to standard systemic therapy alone or in combination with locoregional treatment (radiotherapy or surgery). This trial failed and did not demonstrate a benefit for LR treatment in progression-free survival (PFS). It’s important to note that, only 20% of the patients presented synchronous primary disease and metastatic lesions [14]. However, these conflicting results have not provided any solid support in the management of these patients and the therapeutic approach must be individualized given the lack of sufficient prospective data [15].

Regarding LRT of isolated liver metastases, retrospective trials have been described and liver metastasectomy appears to demonstrate a survival benefit in selected cases. However, there are no data from prospective randomized trials and the level of evidence is scarce [16, 17]. A retrospective study of 86 patients that underwent resection of breast cancer liver metastases showed an improved OS for patients with HR+ BC in response to systemic treatment before surgery [18]. Other local therapies such as radiofrequency (RFA) or microwave ablation (MWA) are also options for such patients [17].

When metastatic breast cancer is diagnosed, even when classified as an oligometastatic disease, systemic treatment is the preferred approach. Previously presented studies usually include patients with different subtypes of breast cancer and have a limited representation of effective systemic treatments. Systemic medical therapy has improved the survival of patients with MBC. In this era of new systemic therapies, such as the CDK4/6 inhibitors (Palbociclib, Ribociclib, and Abemaciclib) in HR+/HER2- BC [19], LR treatments for patients with limited OM disease may become more relevant. These data reinforce the need to understand the role of combination therapy with CDK4/6 inhibitors and locoregional treatment. The ongoing PALATINE trial (NCT03870919), with Palbociclib in HR+/HER- BC, attempts to answer these questions. 

Gion et al., in a recently published review, suggest treating these patients with systemic therapy in a "neoadjuvant-like" strategy, followed by LR treatment and, subsequently, with adjuvant systemic treatment [15]. There is still a need to clarify the best systemic treatment and the most appropriate duration. After finishing the treatment with curative intent, there is no scientific data regarding specific follow-up procedures for these patients. 

## Conclusions

We report a successful clinical case combining systemic treatment and surgical approach in stage IV de novo BC patients with single liver metastasis. Our clinical case suggests that this integrated therapeutic strategy should be considered in highly selected cases after a careful multidisciplinary evaluation. However, several questions regarding OM BC management remain uncertain. The need for well-designed prospective randomized studies is imperative to assist in the selection of patients who can most benefit from this combined strategy and to determine the most appropriate locoregional and systemic treatment. Also, a more comprehensive workout based not only on imaging findings but including other risk factors and biomarkers (such as ctDNA) may improve the selection and consequently the outcomes.
